# Socio-demographic inequalities across a range of health status indicators and health behaviours among pregnant women in prenatal primary care: a cross-sectional study

**DOI:** 10.1186/s12884-015-0676-z

**Published:** 2015-10-13

**Authors:** Ruth Baron, Judith Manniën, Saskia J. te Velde, Trudy Klomp, Eileen K. Hutton, Johannes Brug

**Affiliations:** Department of Midwifery Science, AVAG and the EMGO Institute for Health and Care Research, VU University Medical Centre, P.O. Box 7057, 1007 MB Amsterdam, The Netherlands; Department of Epidemiology and Biostatistics and the EMGO Institute for Health and Care Research, VU University Medical Centre, P.O. Box 7057, 1007 MB Amsterdam, The Netherlands; McMaster University, 1280 Main St. W., MDCL 2210, Hamilton, ON L8S 4 K1 Canada

**Keywords:** Health inequality, Midwifery, Pregnancy, Primary care, Public health

## Abstract

**Background:**

Suboptimal maternal health conditions (such as obesity, underweight, depression and stress) and health behaviours (such as smoking, alcohol consumption and unhealthy nutrition) during pregnancy have been associated with negative pregnancy outcomes. Our first aim was to give an overview of the self-reported health status and health behaviours of pregnant women under midwife-led primary care in the Netherlands. Our second aim was to identify potential differences in these health status indicators and behaviours according to educational level (as a proxy for socio-economic status) and ethnicity (as a proxy for immigration status).

**Methods:**

Our cross-sectional study (data obtained from the DELIVER multicentre prospective cohort study conducted from September 2009 to March 2011) was based on questionnaires about maternal health and prenatal care, which were completed by 6711 pregnant women. The relationships of education and ethnicity with 13 health status indicators and 10 health behaviours during pregnancy were examined using multilevel multiple logistic regression analyses, adjusted for age, parity, number of weeks pregnant and either education or ethnicity.

**Results:**

Lower educated women were especially more likely to smoke (Odds Ratio (OR) 11.3; 95 % confidence interval (CI) 7.6– 16.8); have passive smoking exposure (OR 6.9; 95 % CI 4.4–11.0); have low health control beliefs (OR 10.4; 95 % CI 8.5–12.8); not attend antenatal classes (OR 4.5; 95 % CI 3.5–5.8) and not take folic acid supplementation (OR 3.4; 95 % CI 2.7–4.4). They were also somewhat more likely to skip breakfast daily, be obese, underweight and depressed or anxious. Non-western women were especially more likely not to take folic acid supplementation (OR 4.5; 95 % CI 3.5–5.7); have low health control beliefs (OR 4.1; 95 % CI 3.1–5.2) and not to attend antenatal classes (OR 3.3; 95 % CI 2.0–5.4). They were also somewhat more likely to have nausea, back pains and passive smoking exposure.

**Conclusions:**

Substantial socio-demographic inequalities persist with respect to many health-related issues in medically low risk pregnancies in the Netherlands. Improved strategies are needed to address the specific needs of socio-demographic groups at higher risk and the structures underlying social inequalities in pregnant women.

## Background

Adverse pregnancy outcomes are still very common in Western countries, including the Netherlands [1, 2]. Suboptimal maternal health conditions (such as obesity, underweight, stress and depression [3–8]) and health behaviours (such as smoking, alcohol consumption and unhealthy nutrition [9–12]) have been associated with adverse pregnancy outcomes. Suboptimal health conditions and behaviours are consistently found to be more prevalent among people from lower socio-economic status –for example as indicated by lower levels of education- and immigrant status. Such differences are important determinants of health inequalities in general [13, 14] and during pregnancy [15, 16]. Additionally, pregnancy-related conditions such as nausea, and pelvic pain, generally considered normal in pregnancy, may increase depression in women [17, 18] and could potentially lead to isolation and decreased social support in some migrant groups [19]. Social inequalities in health conditions during pregnancy (such as nausea, back pains and pelvic pains) and health behaviours (such as skipping breakfast and dinner) have previously had little attention. In order to inform and to better tailor and target interventions to promote positive maternal health and pregnancy outcomes, it is important to gain a better insight into the differences in prevalence of suboptimal maternal health indicators and behaviours across social groups.

Suboptimal health and health behaviours may also occur in pregnant women who are considered low risk from a medical point of view. In the Netherlands 84.9 % of women start their pregnancy under the supervision of a primary care midwife with the assumption that they are low risk [20]. Women are referred to secondary care if complications, such as preeclampsia, arise during pregnancy. Women with pre-existing risks for complications, such as diabetes, start their pregnancies in secondary care under the supervision of an obstetrician [21]. This division into primary and secondary care makes it possible to focus research on a relatively similar population without serious medical complications at the start of pregnancy.

The aims of this paper are to provide an overview of self-reported health status and health behaviours of pregnant women in primary care who are living in the Netherlands and to identify potential differences in health status and behaviours according to educational level (as a proxy for socio-economic status) and ethnicity (as a proxy for immigration status).

## Methods

### Recruitment and study population

The data for this study were obtained from the DELIVER study (September 2009 – March 2011), a multicentre prospective cohort study consisting of 7865 low risk pregnant women. DELIVER is an acronym for the Dutch terms **D**ata **E**erste**LI**jns **VER**loskunde, which is translated as Data Primary Care Midwifery. Details about the design of the study can be obtained elsewhere [22]. In brief, twenty midwifery practices distributed throughout the Netherlands were asked to invite their clients for a period of twelve months to complete three questionnaires about various aspects surrounding their pregnancy and health care (before 35 weeks of pregnancy (questionnaire 1); between 35 weeks and birth (questionnaire 2); after giving birth (questionnaire 3)). Before the study began, there was a three-month pilot phase during which the questionnaires were tested among 710 clients and subsequently adjusted if necessary. During the first month of the study period, the practices invited all their clients in various stages of their pregnancy and who had just given birth. In the following months up until the end-date of the study period, new clients were invited to complete the questionnaires, which were online or written (usually starting with questionnaire 1). If the latter part of their pregnancy and labour fell within the study period, the women who had completed questionnaire 1 were also invited to complete questionnaires 2 and 3. This resulted in considerably more respondents who had completed questionnaire 1 than questionnaires 2 and 3. An additional inclusion criterion was understanding at least one of the following languages: Dutch, English, Turkish or Arabic. Non-responders were sent written reminders and called by research assistants if they had not responded within one week. Telephone interviews were offered to Turkish and Arabic speaking women who had not responded to the initial invitation. The overall net response of those who had completed at least one of the three questionnaires was 62 %. All women who participated in the study gave their informed consent to their midwives. Ethical approval was obtained for this study from the Medical Ethics Committee of the VU University Medical Centre in Amsterdam on December 9^th^, 2009 (Ref. 2009/284).

For the current study, we used data obtained from the first and second questionnaires, as these were the questionnaires completed during pregnancy. The sample therefore consisted of women who had either completed questionnaire 1 only, questionnaire 2 only or both questionnaires. We included women whose information was complete about their educational level, ethnicity, age, parity and number of weeks of pregnancy at the time of questionnaire completion.

### Study measures

As the two questionnaires contained different health-related questions, cross-sectional analyses were performed on the data of each questionnaire separately. The variables were categorized into two groups broadly defined as either ‘health status variables’ or ‘health behaviour variables’ (see Table 1).

**Table 1:** The columns show the variables selected from questionnaire 1 and from questionnaire 2 for our study

Questionnaire 1	Questionnaire 2
(up to 34 weeks of pregnancy)	(35 weeks till birth)
*N* = 6021	*N* = 3417
Independent variables/potential confounders	
Socio-demographic variables:	Socio-demographic variables:
- Education	- Education
- Ethnicity	- Ethnicity
- Age	- Age
- Parity	- Parity
- No. of weeks pregnant Q1	- No. of weeks pregnant Q2
Dependent variables	
Health status variables:	Health status variables:
- General health	- Health complaints
- Daily functioning	- Nausea ≤ 20 weeks
- Mobility	- Nausea > 20 weeks
- Pain/other complaints	- Fatigue ≤ 20 weeks
- Depression/anxiety	- Fatigue > 20 weeks
- Chronic disease /handicaps	- Dizziness ≤ 20 weeks
- Health control beliefs	- Dizziness > 20 weeks
- Weight status	- Back pain ≤ 20 weeks
	- Back pain > 20 weeks
	- Pelvic pains ≤ 20 weeks
	- Pelvic pains > 20 weeks
Health behaviour variables:	Health behaviour variables:
- Smoking	- Antenatal class attendance
- Passive smoking	
- Alcohol consumption	
- Folic acid supplementation	
- Pregnancy planning	
- Daily fresh vegetable consumption	
- Daily fruit consumption	
- Daily hot meal	
- Daily breakfast	

Women had completed either questionnaire 1, questionnaire 2 or both questionnaires. The first column contains the variables selected from questionnaire 1 and the second column contains the variables selected from questionnaire 2. The questionnaire populations were treated as separate study populations and analyzed separately

#### *Socio-demographic variables*

Questionnaires 1 and 2: Respondents were asked to complete their socio-demographic details only once, irrespective of which and how many questionnaires they had completed. They were asked to indicate their highest completed educational level. Education was categorized as ‘high’ (college, university or post-graduate education), ‘mid-level’ (secondary school, or mid-level vocational education) and ‘low’ (lower vocational education or less). A variable ‘ethnicity’ was created with the three categories (‘Dutch’, ‘western ethnic minority’ or ‘non-western ethnic minority’), according to the definitions of Statistics Netherlands [23]. Women were considered to be of western ethnicity if at least one of their parents was born in North America, Europe (except for Turkey), Oceania, Japan or Indonesia; women were considered to be of non-western ethnicity if at least one of their parents was born in Turkey, Africa, Asia (except for Japan and Indonesia) or South America. The variables ‘age’, ‘parity’ and ‘numbers of weeks pregnant’ were included as possible confounders. Age (derived from date of birth) and current weeks of pregnancy as reported by respondents, were both continuous variables. The dichotomous variable ‘parity’ was based on a question asking the respondents how many children they already had (‘nulliparous’ (no children) vs. ‘multiparous’ (at least one child)).

#### *Health status variables*

Questionnaire 1 (before 35 weeks of pregnancy): Respondents were asked to describe their ‘general health’ and ‘daily functioning’ at home, work, and in their free time with five response options ranging from excellent to poor. For both questions, we dichotomized these options into ‘(very) good/excellent’ and ‘mediocre/poor’. Respondents were asked whether they had any chronic diseases or disabilities with response options ‘yes’ or ‘no’. Those who had indicated having a chronic disease were then asked by means of an open-ended question to report any chronic diseases they had. ‘Weight status’ was based on self-reported weight and height at the beginning of pregnancy: ‘underweight’ (<18.5 kg/m2), ‘normal weight’ (18.5–24.99 kg/m2), ‘overweight’ (25–29.99 kg/m2) and ‘obese’ (≥30 kg/m2). The health control beliefs variable was based on the question to what extent respondents believed they could control their health by their own behaviours. The four response options ranging from ‘very much so’ to ‘not at all’ were dichotomized into 'very much/quite a bit’ and ‘hardly/not at all’.

Respondents were asked about their current mobility (being able to walk) obtained from the validated EuroQol questionnaire on self-perceived health [24]; the three response options were dichotomized into ‘no difficulties’ and ‘some difficulties/bedridden’. Another item from the EuroQol asked about their current mood and had three possible response options that were dichotomized into ‘not anxious or depressed’ and ‘somewhat/very anxious or depressed’. The final EuroQol item used in this study asked about pain or other complaints and had three response options that were dichotomized into ‘no pain or other complaints’ and ‘some/severe pain or other complaints’.

Questionnaire 2 (between 35 weeks and birth): Respondents were asked to indicate whether they had experienced any health complaints in the first 20 weeks of pregnancy, leading them to have to take things easy. This question had response options ‘yes’ or ‘no’. Those who responded ‘yes’ could indicate in a list of health complaints (‘nausea’, ‘fatigue’, ‘dizziness’, ‘back pains’, ‘pelvic pains’ and ‘other’) which complaints they had experienced. Each health complaint was dichotomized into ‘yes’ or ‘no’. If they indicated ‘other’, they were asked to report which health complaints these were. These health complaint questions were subsequently repeated for the period after 20 weeks of pregnancy.

#### *Health behaviour variables*

Questionnaire 1 (before 35 weeks of pregnancy): Respondents were asked if they currently smoked with response options ‘yes, daily’, ‘yes, occasionally’ and ‘no’. The options ‘yes, daily’ and ‘yes, occasionally’ were combined to form the variable ‘smoking’ with response options ‘yes’ or ‘no’. Passive smoking, with response options ‘yes’ and ‘no’, was created from two questions, asking if there was anyone who smoked in their home and if the respondent herself smoked. If someone smoked in her home, but the respondent herself did not, this was categorized as ‘yes’ (‘passive smoker’). The response option ‘no’ was designated to respondents who indicated being non-smokers as well as having no one who smoked in their homes. Alcohol consumption was based on the question asking respondents if they had consumed any alcoholic drink since knowing they were pregnant, with response options ‘yes’ and ‘no’. Respondents were asked if they had taken/were taking folic acid due to this pregnancy with response options ‘yes’ or ‘no’. They were also asked whether their pregnancy was planned or not, with response options ‘yes’ or ‘no’. Finally, respondents were asked various questions about their diet: whether or not they consumed fresh vegetables, fruit, and a hot meal, each on a daily basis, with response options ‘yes’ or ‘no’. They were also asked how often they ate breakfast per week. The four possible response options were dichotomized into ‘daily’ versus ‘not daily’.

Questionnaire 2 (before 35 weeks of pregnancy): Respondents who completed questionnaire 2 were asked if they had participated or were participating in any antenatal classes, with response options ‘yes’ or ‘no’. Examples of antenatal classes were ‘yoga’, ‘pregnancy gymnastics’ and educational courses in the Netherlands such as ‘Giving birth together’.

### Statistical analyses

Descriptive statistics of socio-demographic variables were run for the data pertaining to each questionnaire separately to give an indication of the representativeness of our study population compared to the general Dutch population. Cross-sectional analyses were carried out on the data from each questionnaire separately to obtain crude frequencies of health status and health behaviour variables, according to education and ethnicity. As our respondents came from 20 different midwife practices, multiple logistic regression using Generalized Estimating Equations (GEE) was used for all dichotomous dependent variables to adjust for possible correlations within practices. Generalized Linear Mixed Models (GLMM) was used for the multinomial dependent variable ‘weight status', as this was not possible with GEE. The association of education (reference category ‘high’) with each dependent variable was adjusted for ethnicity, age, parity and number of weeks pregnant. The association of ethnicity (reference category ‘Dutch’) with each dependent variable was adjusted for education, age, parity and number of weeks pregnant. The results were quantified in odds ratios and 95 % confidence intervals. As this study involved multiple testing and therefore an increase in the chance of type I errors, we interpreted small odds ratios with caution and took clinical relevance into consideration. All analyses were carried out in IBM SPSS version 20.

## Results

**Fig. 1:**
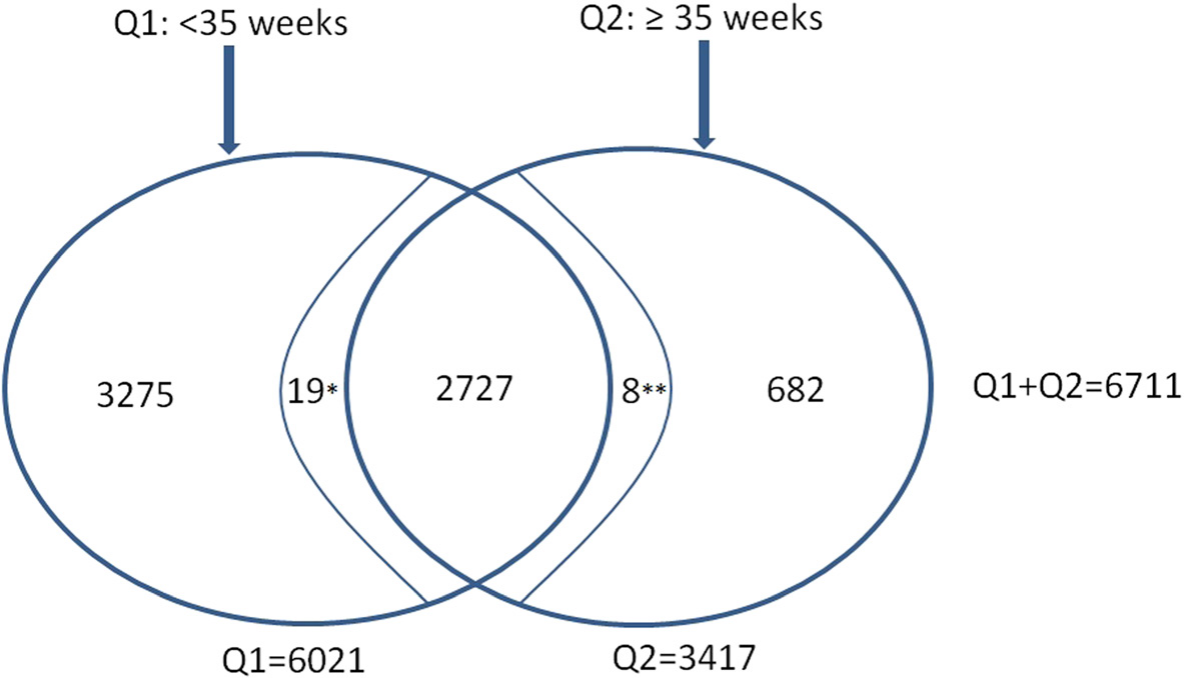
Distribution of respondents who completed questionnaire 1 (Q1) and questionnaire 2 (Q2). The overlapping area between the two oval circles contains the number of respondents who were both part of the sample who completed Q1, as well as the sample who completed Q2. *Completed Q1 & Q2, but due to missing data were not included in the analyses of the Q2 sample. **Completed Q1 & Q2, but due to missing data were not included in the analyses of the Q1 sample

The data for 6711 women were used for analyses, with 6021 women responding to questionnaire 1 and 3417 to questionnaire 2. There was an overlap of 2727 women between the two questionnaires (see Fig. 1).

### Socio-demographics

Questionnaire 1: the average age was 30.4 years (SD 4.6), 45.5 % were nulliparous and 54.5 % were multiparous. The proportion of highly educated women was 48.8 %, and the proportion of ethnic minority groups was 16.2 % (western 7.6 % and non-western 8.6 %). Questionnaire 2: the average age was 30.8 years (SD 4.5), 46.3 % were nulliparous and 53.7 % were multiparous. The proportion of highly educated women was 52.6 % and the proportion of ethnic minority groups was 13.5 % (western 7.2 % and non-western 6.3 %). Age and parity of both study samples were comparable to the general population who had given birth in the Netherlands in 2010 (average age:31.0 (SD 5.0); nulliparity: 47.5 %, multiparity: 52.5 % [25]). There were more highly educated women in both samples and fewer women from ethnic minority groups compared to the general population of women between 15 and 54 years of age (28.2 % and 22.7 % respectively, 9.6 % western and 13.1 % non-western) [23]. The median number of weeks of pregnancy was 19 for questionnaire 1 and 37 for questionnaire 2.

### Health status variables (Table 2)

**Table 2:** Proportions of unbeneficial health status indicators according to education and ethnicity. Odds ratios and 95 % confidence intervals of health status variables according to education and ethnicity, adjusted for parity, age and number of weeks pregnant using Generalized Estimating Equations (GEE)

Unbeneficial health status indicators	Proportion of total sample	By education^c^					By ethnicity^b^				
		High (Ref)	Mid-level		Low		Dutch (Ref)	Western ethnic minority		Non-Western ethnic minority	
	*N* (%)	*N* (%)	*N* (%)	OR (95 % CI)	*N* (%)	OR (95 % CI)	*N* (%)	*N* (%)	OR (95 % CI)	*N* (%)	OR (95 % CI)
Low Health control Beliefs	908/5996 (15.1)	143 (4.9)	399 (18.5)	**3.8 (3.1–4.7)**	366 (40.4)	**10.4 (8.5–12.8)**	641 (12.7)	57 (12.6)	1.1 (0.8–1.4)	210 (41.2)	**4.1 (3.1–5.2)**
General health Mediocre/poor	733/6000 (12.2)	325 (11.1)	269 (12.4)	1.1 (0.9–1.3)	139 (15.3)	1.3 (1.0–1.7)	581 (11.5)	57 (12.5)	1.1 (0.9–1.5)	95 (18.5)	**1.7 (1.3–2.1)**
Daily functioning Mediocre/ poor	1191/5998 (19.9)	529 (18.1)	449 (20.7)	1.2 (1.0–1.3)*	213 (23.5)	1.3 (1.0–1.6)*	960 (19.1)	96 (21.1)	1.2 (0.9–1.4)	135 (26.3)	**1.4 (1.2 –1.7)**
Somewhat/very depressed/ anxious mood	1194/6004 (19.9)	459 (15.7)	464 (21.4)	**1.4 (1.2 –1.6)**	271 (29.9)	**2.0 (1.7–2.3)**	915 (18.2)	100 (22.0)	**1.3 (1.1–1.6)**	179 (34.8)	**2.2 (1.7–2.9)**
Mobility: some difficulty/bedridden	1219/6004 (20.3)	512 (17.5)	481 (22.2)	**1.3 (1.1–1.5)**	226 (24.9)	**1.3 (1.1–1.6)**	968 (19.2)	96 (21.1)	1.2 (0.9–1.5)	155 (30.1)	**1.6 (1.3–2.1)**
Pains/other complaints	3163/6003 (52.7)	1385 (47.3)	1210 (55.9)	**1.3 (1.1–1.5)**	566 (62.4)	**1.6 (1.4–1.9)**	2556 (50.8)	263 (57.9)	**1.4 (1.2–1.6)**	344 (66.8)	**1.7 (1.4–2.0)**
Chronic disease	605/6003 (10.1)	249 (8.5)	255 (11.8)	**1.5 (1.2 –1.8)**	101 (11.1)	**1.4 (1.1–1.8)**	515 (10.2)	47 (10.4)	1.0 (0.7–1.4)	43 (8.3)	0.7 (0.5–1.0)
Weight status^a^											
Underweight	189/5640 (3.4)	80 (2.9)	70 (3.5)	1.3 (1.0–1.6)	39 (4.8)	**1.9 (1.3–2.6)**	153 (3.2)	19 (4.4)	1.4 (0.9–2.2)	17 (3.7)	1.1 (0.5–2.6)
Overweight	1229/5640 (21.8)	534 (19.0)	476 (23.5)	**1.4 (1.2–1.6)**	219 (27.1)	**1.6 (1.4–2.0)**	1019 (21.5)	78 (18.3)	0.9 (0.7–1.1)	132 (28.5)	**1.4 (1.1–1.8)**
Obese	438/5640 (7.8)	139 (5.0)	213 (10.5)	**2.4 (1.9–3.0)**	86 (10.7)	**2.6 (1.8–3.8)**	366 (7.7)	35 (8.2)	1.1 (0.8–1.4)	37 (8.0)	1.1 (0.8–1.4)
Health complaints ≤ 20 weeks	2197/3332 (65.9)	1144 (65.2)	754 (65.4)	1.0 (0.8–1.2)	299 (70.5)	1.2 (0.9–1.5)	1864 (64.6)	175 (73.8)	**1.6 (1.3–2.0**)	158 (75.6)	**1.6 (1.3–2.0)**
Health complaints >20 weeks	2274/3330 (68.3)	1163 (66.3)	818 (70.9)	1.2 (1.0–1.4)	293 (69.3)	1.1 (0.9–1.3)	1955 (67.8)	174 (73.4)	1.4 (1.0–1.8)*	145 (69.7)	1.0 (0.8-1.4)
Nausea ≤ 20 weeks	1327/3332 (39.8)	703 (40.1)	431 (37.4)	0.8 (0.7–1.0)	193 (45.5)	1.1 (1.0–1.3)	1097 (38.0)	121 (51.1)	**1.7 (1.4–2.2)**	109 (52.2)	1.7 (1.3–2.2)*
Nausea >20 weeks	273/3330 (8.2)	128 (7.3)	103 (8.9)	1.1 (0.9–1.4)	42 (9.9)	1.1 (0.8–1.6)	215 (7.5)	18 (7.6)	1.1 (0.6–1.9)	40 (19.2)	**2.7 (1.9–4.0)**
Fatigue ≤ 20 weeks	1487/3332 (44.6)	817 (46.6)	483 (41.9)	0.9 (0.7–1.0)	187 (44.1)	0.9 (0.7–1.2)	1269 (44.0)	120 (50.6)	1.3 (1.0–1.7)	98 (46.9)	1.1 (0.9–1.5)
Fatigue > 20 weeks	1221/3330 (36.7)	642 (36.6)	425 (36.9)	1.0 (0.9–1.2)	154 (36.4)	1.0 (0.8–1.3)	1046 (36.3)	98 (41.4)	1.2 (1.0–1.5)*	77 (37.0)	1.0 (0.8–1.3)
Dizziness ≤ 20 weeks	372/3332 (11.2)	177 (10.1)	135 (11.7)	1.0 (0.8–1.4)	60 (14.2)	1.1 (0.8–1.6)	293 (10.2)	30 (12.7)	1.4 (1.0–1.9)	49 (23.4)	**2.5 (1.9–3.2)**
Dizziness > 20 weeks	286/3330 (8.6)	130 (17.4)	103 (8.9)	1.2 (0.9–1.5)	53 (12.5)	**1.6 (1.2–2.3)**	245 (8.5)	14 (5.9)	0.7 (0.4–1.1)	27 (13.0)	1.4 (0.8–2.3)
Back pain ≤ 20 weeks	352/3332 (10.6)	157 (8.9)	135 (11.7)	1.2 (0.9–1.6)	60 (14.2)	1.3 (1.0–1.7)*	274 (9.5)	28 (11.8)	1.4 (0.9–1.9)	50 (23.8)	**2.7 (2.0–3.7)**
Back pain >20 weeks	799/3330 (24.0)	354 (20.2)	320 (27.8)	**1.4 (1.1–1.8)**	125 (29.6)	**1.5 (1.2–1.8)**	666 (23.1)	68 (28.7)	1.4 (1.0–2.0)	65 (31.2)	**1.4 (1.1–1.8)**
Pelvic pain ≤ 20 weeks	466/3332 (14.0)	209 (11.9)	193 (16.7)	1.3 (1.0–1.7)*	64 (15.1)	1.1 (0.8–1.5)	395 (13.7)	33 (13.9)	1.1 (0.8–1.7)	38 (18.2)	1.3 (1.0–1.7)*
Pelvic pain >20 weeks	942/3330 (28.3)	476 (27.1)	348 (30.2)	1.1 (0.8–1.3)	118 (27.9)	0.9 (0.7–1.2)	800 (27.7)	85 (35.9)	**1.5 (1.2–2.1)**	57 (27.4)	0.9 (0.7–1.2)

Ref: reference category

Odds ratios in bold are significant

^**a**^Generalized Linear Mixed Models (GLMM) used for the multinomial variable weight status

*rounding error: *p* = <0.05

^b^adjusted for ethnicity, parity, age and number of weeks pregnant

^c^adjusted for education, parity, age and number of weeks pregnant

Most women rated their general health and daily functioning positively, with 12.2 % and 19.9 % respectively, rating these as mediocre or poor. About two thirds of all women (65.9 % up to 20 weeks of pregnancy and 68.3 % after 20 weeks of pregnancy) indicated having health complaints, of which the most prevalent were pain/other complaints (52.7 %), fatigue ≤ 20 weeks and > 20 weeks (44.6 % and 36.7 %), nausea ≤ 20 weeks (39.8 %), and pelvic and back pains > 20 weeks of pregnancy (28.3 % and 24.0 % respectively). The ‘other’ complaints up to 20 weeks most frequently mentioned were blood loss, Braxton Hicks contractions of the uterus, headaches and psychological problems (eg. stress or depression). The most frequently mentioned ‘other’ complaints after 20 weeks of pregnancy were Braxton Hicks contractions of the uterus, heartburn, high blood pressure, water retention and nerve pains such as sciatica.

Almost one third (29.6 %, *N* = 1667) of women were overweight or obese at the start of their pregnancy, 20.3 % (1219) had some difficulty walking or were bedridden and 19.9 % (1194) currently felt somewhat to very depressed or anxious. Having a chronic disease or handicap was reported by 10.1 % (605) of women; the most frequently mentioned were asthma, thyroid problems, arthritic diseases (including fibromyalgia), pelvic and back complaints, psychological problems and inflammatory intestinal complaints.

#### *Health status according to educational level*

Low health control beliefs were ten times more likely in women with low education and almost four times more likely in those with mid-level education, compared to those with high education. Obesity was more than twice as likely in those with low and mid-level education and depressed mood or anxiety was twice as likely in women with low and somewhat more likely in those with mid-level education. Overweight (not including obesity), pains or other complaints, poor mobility, back pains throughout pregnancy, chronic diseases or handicaps and mediocre to poor daily functioning were somewhat more likely in women of low and mid-level education compared to those of high education.

Comparable across educational levels were general health, total health complaints throughout pregnancy, fatigue throughout pregnancy, nausea throughout pregnancy, dizziness (≤20 weeks) and pelvic pains (>20 weeks).

#### *Health status according to ethnicity*

The health status variable with the largest difference in ethnic minority groups was health control beliefs, with non-western ethnic minorities being four times more likely to have low health control beliefs than those of Dutch ethnicity. Back pains (≤20 weeks) and nausea (>20 weeks) were 2.7 times more likely, dizziness (≤20 weeks) 2.5 times more likely and depression or anxiety twice as likely in non-western ethnicities. They were somewhat more likely to rate their general health as well as their general functioning as mediocre or poor, to be overweight (BMI 25–29.99), to report having general health complaints, back pains (>20 weeks), pain/other complaints and poor mobility. Western ethnic minorities were somewhat more likely than those of Dutch ethnicity to report general health complaints, pains/other complaints, depression or anxiety, nausea (≤20 weeks) and pelvic pains (>20 weeks).

Western minorities were comparable to those of Dutch ethnicity with respect to low health control beliefs, general health, daily functioning, mobility, chronic disease or handicaps, all weight categories, fatigue, nausea (>20 weeks), dizziness and back pains throughout pregnancy and pelvic pains (≤20 weeks). Non-western minorities were comparable to Dutch ethnicity in chronic disease or handicaps, underweight and obesity, fatigue throughout pregnancy, dizziness (>20 weeks) and pelvic pains (>20 weeks).

### Health behaviour variables (Table 3)

**Table 3:** Proportions of unbeneficial health behaviours according to education and ethnicity. Odds ratios and 95 % confidence intervals of health behaviour characteristics according to education, ethnicity and adjusted for parity, age and number of weeks pregnant, using multiple logistic regression with Generalized Estimating Equations (GEE)

Unbeneficial health behaviours	Proportion of total sample	By education^b^					By ethnicity^a^				
		High (Ref)	Mid-level		Low		Dutch (Ref)	Western ethnic minority		Non-Western ethnic minority	
	*N* (%)	*N* (%)	*N* (%)	OR (95 % CI)	*N* (%)	OR (95 % CI)	*N* (%)	*N* (%)	OR (95 % CI)	*N* (%)	OR (95 % CI)
Smoking	553/6002 (9.2)	77 (2.6)	249 (11.5)	**4.3 (3.2–5.9)**	227 (25.1)	**11.3 (7.6–16.8)**	454 (9.0)	48 (10.5)	1.3 (0.9–1.7)	51 (9.9)	0.7 (0.5–1.0)
Passive Smoking	285/5449 (5.2)	57 (2.0)	134 (7.0)	**3.2 (2.3–4.5)**	94 (13.8)	**6.9 (4.4–11.0)**	207 (4.5)	24 (5.9)	1.4 (0.8–2.2)	54 (11.6)	**2.1 (1.5–3.1)**
No folic acid supplementation	515/6005 (8.6)	150 (5.1)	183 (8.4)	**1.6 (1.2–2.1)**	182 (20.1)	**3.4 (2.7–4.4)**	330 (6.6)	41 (9.0)	**1.5 (1.1–2.1)**	144 (28.0)	**4.5 (3.5–5.7)**
Alcohol consumption	659/5995 (11.0)	404 (13.8)	188 (8.7)	**0.7 (0.6–0.8)**	66 (7.4)	**0.6 (0.5–0.9)**	568 (11.3)	59 (13.0)	1.0 (0.7–1.6)	32 (6.2)	0.6 (0.3–1.0)*
Unplanned pregnancy	1057/6011 (17.6)	378 (12.9)	423 (19.5)	**1.5 (1.3–1.7)**	256 (28.3)	**2.2 (1.9–2.5)**	818 (16.2)	97 (21.3)	**1.5 (1.2–1.9)**	142 (27.6)	**1.5 (1.3-1.9)**
No antenatal class attendance	1812/3338 (54.3)	768 (43.6)	713 (61.6)	**2.1 (1.8–2.5)**	333 (78.5)	**4.5 (3.5–5.8)**	1518 (52.5)	132 (55.5)	1.4 (1.0–1.9)*	164 (78.5)	**3.3 (2.0–5.4)**
No daily vegetables	1116/5995 (18.6)	461 (15.7)	467 (21.6)	1.3 (1.0–1.6)*	188 (20.8)	1.2 (1.0–1.5)	948 (18.9)	61 (13.4)	0.7 (0.5–1.0)*	107 (20.8)	1.1 (0.9–1.3)
No daily fruit consumption	879/5996 (14.7)	350 (12.0)	342 (15.8)	1.2 (1.0–1.5)*	187 (20.7)	**1.7 (1.3–2.1)**	743 (14.8)	60 (13.2)	0.9 (0.7–1.2)	76 (14.8)	0.9 (0.7–1.0)
No daily hot meal	189/5995 (3.2)	74 (2.5)	83 (3.8)	**1.6 (1.2–2.2)**	32 (3.5)	**1.5 (1.1–2.1)**	151 (3.0)	20 (4.4)	1.5 (0.8–2.7)	18 (3.5)	1.1 (0.7–1.7)
No daily breakfast	666/5993 (11.1)	209 (7.1)	283 (13.1)	**1.7 (1.4–2.0)**	174 (19.2)	**2.5 (2.1–3.1)**	525 (10.4)	47 (10.3)	1.0 (0.7–1.5)	94 (18.3)	1.5 (0.9–2.3)

Ref: reference category

Odds ratios in bold are significant

*rounding error: *p* = <0.05

^a^adjusted for ethnicity, parity, age and number of weeks pregnant

^b^adjusted for education, parity, age and number of weeks pregnant

More than half of all women (54.3 %, *N* = 1812) did not attend an antenatal class. Regarding nutrition, 18.6 % (1116) of women reported not eating fresh vegetables daily, 14.7 % (879) not eating fruit daily, 11.1 % (666) not having breakfast daily and 3.2 % (189) did not eat a hot meal daily. The pregnancy was unplanned for 17.6 % (1057) of women.

Smoking was reported by 9.2 % (553) of women and passive smoking by 5.2 % (285) of non-smoking women. Folic acid was not taken at all during the current pregnancy by 8.6 % (515) of women and an alcoholic drink was consumed at least once by 11.0 % (659) of pregnant women.

#### *Health behaviours according to educational level*

All health behaviour variables showed at least some disparity across the educational levels. The largest disparity was seen in smoking, which was 11 times more likely in low and four times more likely in those of mid-level education compared to high education. Passive smoking was seven times more likely in low and three times more likely in those of mid-level education. Not attending antenatal classes was 4.5 times more likely in women of low and twice as likely in those of mid-level education. Not taking a folic acid supplement was three times more likely in those of low education and somewhat more likely in those of mid-level education. Unplanned pregnancy, skipping breakfast, no hot meal and no fruit consumption on a daily basis were more likely in women with mid-level and low education. Alcohol consumption was less likely in those of mid-level and low education, compared to those of high education.

The daily consumption of fresh vegetables was comparable across educational levels.

#### *Health behaviours according to ethnicity*

About half of all health behaviours showed disparities across ethnic groups. Non-western ethnic minorities were 4.5 times more likely to not take folic acid supplements, three times more likely to not attend antenatal classes, twice as likely to have passive smoking exposure, somewhat more likely to have had an unplanned pregnancy than those of Dutch ethnicity. They were also somewhat less likely to have consumed alcohol during pregnancy. Western ethnic minorities were somewhat less likely to have taken folic acid, to attend an antenatal class and more likely to have had an unplanned pregnancy. They were slightly more likely to consume fresh vegetables daily than those of Dutch ethnicity.

Western minorities were comparable to those of Dutch ethnicity in smoking, passive smoking, alcohol consumption and daily fruit, hot meal and breakfast consumption. Non-western minorities were comparable to Dutch ethnicity in smoking and daily vegetable, fruit, hot meal and breakfast consumption.

## Discussion

Our study aimed to assess the health status and health behaviours of pregnant women in primary care. Although the vast majority of women rated their general health as good to excellent, two-thirds of all women indicated having health complaints.

There were many sizable disparities in unbeneficial health status indicators and health behaviours according to educational level and ethnicity. In educational level these were low health control beliefs, obesity and underweight, depression/anxiety, smoking, passive smoking exposure, no antenatal class attendance, no folic acid supplementation, skipping breakfast daily, unplanned pregnancy and no daily fruit consumption. In ethnicity these were low health control beliefs, depression/anxiety, back pain and dizziness (≤20 weeks), nausea (>20 weeks), no folic acid supplementation, no antenatal class attendance and passive smoking exposure.

The most prominent health status indicator showing differences across both education level and ethnicity was low health control beliefs. Low health control means that one believes that health is influenced by causes outside of their own control. This perceived lack of control may be due to the higher rate of detrimental health issues they may experience. Additionally, facing daily struggles, such as the stresses associated with low income, has been found to negatively influence such control beliefs [26]; women from more vulnerable groups, such as those with lower incomes or certain migrant groups are more likely to have to deal with such daily struggles [19]. This may reflect the ‘fundamental social causes’ theory, which posits that as health and illness potentially come more under the control of people through biomedical knowledge and technology, social inequalities increase because of the unequal distribution of this control [27]. An advisory committee for the Ministry of Health, which was assembled to reduce social inequalities in the Netherlands in 2001 also recognized that individuals do not have complete control over their health and health behaviours [28]. In our study, low health control beliefs may be related to all the other health status indicators and behaviours in which social inequality is apparent.

Our study showed that women with lower education were more likely to be obese or to be underweight. Differences in nutritional consumption, physical activity and meal and smoking patterns are likely to be contributing causes. Earlier studies have reported the relationship of education with pre pregnancy obesity [29, 30]; reports on socio-demographic factors associated with pre pregnancy underweight are scarce, however, possibly due to underweight not being considered a real health issue in high income countries. Women of mid and low education as well as non-western ethnic minority more frequently reported pains or other complaints, poor mobility and especially back pains, which are similar to the findings of other studies and may be associated with occupation type [31].

In our study, the most prominent health behaviours with educational disparities were smoking, followed by passive smoking, which is in line with previous studies [32, 33]. Smokers with higher education are more likely to stop smoking, upon finding out they are pregnant [34] leading in turn to more disparity in smoking. Smoking during pregnancy may be an important mediator between low education and various adverse perinatal outcomes [35, 36]. Major health gains may therefore be achieved by making smoking cessation a priority in perinatal health promotion.

Our study also showed inequalities in antenatal class attendance, possibly reflecting earlier findings that lower educated groups and immigrants are less likely to seek (extra) health care [31, 37, 38]. They may be missing an opportunity to be exposed to maternal health promotion and to be in contact with other pregnant women for social support, which in turn is associated with pregnancy outcomes [39].

Similar to our study, earlier studies have reported the relationship of educational level with health behaviours during pregnancy such as folic acid supplementation [40], unplanned pregnancy [41] and daily fruit consumption (22). Skipping breakfast was also more likely in those of lower education in our study and may be a proxy for other factors associated with those groups. The only health behaviour more favourable among both lower educated and non-western ethnic minority women was no alcohol consumption. A higher mean alcohol intake among higher educated people in the Netherlands has also been reported for the general population [42].

Awareness of social inequalities in health has been present for decades, and although efforts carried in the Netherlands have made progress in reducing the gap between social groups in general [13], our findings confirm that social inequalities in health continue to persist in pregnancy. Theories proposed for this persistence of health inequalities include inadequate income redistribution, health inequalities being more related to immaterial factors such as cultural factors, and people of higher socio-economic status benefiting relatively more from improvements in healthcare than people of lower socio-economic status [43].

As pregnancy may be the only time that many women have regular contact with health care providers, such as midwives, this is an opportunity to help increase the quality of life for women and their families beyond the care of their pregnancies. A report entitled ‘A Good Beginning’ (2010) written by an advisory committee for the Ministry of Health in the Netherlands to improve perinatal health and reduce inequalities, underlined the importance of screening women for risks related to poverty, lifestyle and psychosocial factors besides medical risks [44]. A greater understanding by prenatal health care providers of the non-medical risks of adverse pregnancy outcomes may benefit those social groups at greater risk. Continued training in cultural differences, assessing and responding to different levels of health literacy in clients, building empathic and trusting relationships, conveying to clients a sense of personal control over their health, and keeping up-to-date with research on health and health promotion, may help to reduce health inequalities [37, 45–47]. Additionally, increased strategies should be employed to target the social determinants of health inequality, such as forming stronger relationships with other relevant branches in housing, employment, social work and working in multidisciplinary teams with other health fields such as nutrition and physiotherapy [45, 47].

### Limitations and strengths

While interpreting the findings, we acknowledge some limitations. Health status and behaviours were self-reported in this study, and bias may have occurred if women provided socially desirable answers despite knowing their contributions would be made anonymous. Our study also had relatively more respondents of high education and of Dutch ethnicity than the general population of pregnant women in the Netherlands. There were enough respondents, however, to be able to identify differences between the various socio-demographic groups. We also divided ethnic minority groups into western and non-western, which does not do justice to differences between first and second-generation minority groups or between specific ethnic groups.

Including the relationships of various health indicators with pregnancy outcomes may have been illustrative; however, due to the large number of variables, we chose to focus on the maternal health indicators during pregnancy. Similarly studying how various health issues are related to each other and to socio-demographics was also beyond the scope of this study. Studies have found that those believing in destiny (related to having low health beliefs), for instance, are more likely to smoke during pregnancy [48] and not to take folic acid [49]. The role of health control beliefs could be elucidated further by mediation analyses. Our study, therefore, however, hopes to lay a foundation for more research into these complex interactions of socio-demographics and health issues.

The main strengths of our study lie in the large study population recruited throughout the Netherlands and the broad range of health indicators. As all women were starting out in prenatal primary care, our study enabled us to provide an insight into potential health gains to be achieved in uncomplicated pregnancies.

## Conclusions

Our study shows notable differences between women of low, mid and high levels of education, as well as between women from non-western ethnic minority groups and Dutch ethnicity in a wide range of health issues during pregnancy, such as low health control beliefs, smoking, no antenatal class attendance, no folic acid supplementation, depression or anxiety, obesity, underweight, skipping breakfast daily and back pains. Improved strategies are needed to address the specific needs of socio-demographic groups at higher risk and the structures underlying social inequalities in pregnant women.
